# Parental Assessment of Postsurgical Pain in Infants at Home Using Artificial Intelligence–Enabled and Observer-Based Tools: Construct Validity and Clinical Utility Evaluation Study

**DOI:** 10.2196/64669

**Published:** 2024-12-03

**Authors:** Fatos Sada, Paola Chivers, Sokol Cecelia, Sejdi Statovci, Kujtim Ukperaj, Jeffery Hughes, Kreshnik Hoti

**Affiliations:** 1 Faculty of Medicine University of Prishtina Prishtina Kosovo; 2 School of Medical and Health Sciences Edith Cowan University Joondalup Australia; 3 Research Department Child and Adolescent Health Service Nedlands Australia; 4 Institute for Health Research The University of Notre Dame Fremantle Australia; 5 Kavaja Hospital Prishtina Kosovo; 6 UBT-Higher Education Institution Prishtina Kosovo; 7 Clinic of Pediatric Surgery University Clinical Center of Kosovo Prishtina Kosovo; 8 Curtin Medical School Curtin University Bentley Australia

**Keywords:** PainChek Infant, Observer-Administered Visual Analog Scale, parents, infant pain, pain assessment, circumcision, infant home assessment, clinical utility, construct validity, artificial intelligence

## Abstract

**Background:**

Pain assessment in the infant population is challenging owing to their inability to verbalize and hence self-report pain. Currently, there is a paucity of data on how parents identify and manage this pain at home using standardized pain assessment tools.

**Objective:**

This study aimed to explore parents’ assessment and intervention of pain in their infants at home following same-day surgery, using standardized pain assessment tools.

**Methods:**

This prospective study initially recruited 109 infant boys undergoing circumcision (same-day surgery). To assess pain at home over 3 days after surgery, parents using iOS devices were assigned to use the PainChek Infant tool, which is a point-of-care artificial intelligence–enabled tool, while parents using Android devices were assigned to use the Observer-Administered Visual Analog Scale (ObsVAS) tool. Chi-square analysis compared the intervention undertaken and pain presence. Generalized estimating equations were used to evaluate outcomes related to construct validity and clinical utility. Receiver operating characteristic analysis assessed pain score cutoffs in relation to the intervention used.

**Results:**

A total of 69 parents completed postsurgery pain assessments at home and returned their pain diaries. Of these 69 parents, 24 used ObsVAS and 45 used PainChek Infant. Feeding alone and feeding with medication were the most common pain interventions. Pain presence over time reduced. In the presence of pain, an intervention was likely to be administered (*χ^2^*_2_=21.4; *P*<.001), with a medicinal intervention being 12.6 (95% CI 4.3-37.0; *P*<.001) times more likely and a nonmedicinal intervention being 5.2 (95% CI 1.8-14.6; *P*=.002) times more likely than no intervention. In the presence of intervention, score cutoff values were ≥2 for PainChek Infant and ≥20 for ObsVAS. A significant effect between the use of the pain instrument (*χ^2^*_1_=7.2, *P*=.007) and intervention (*χ^2^*_2_=43.4, *P*<.001) was found, supporting the construct validity of both instruments. Standardized pain scores were the highest when a medicinal intervention was undertaken (estimated marginal mean [EMM]=34.2%), followed by a nonmedicinal intervention (EMM=23.5%) and no intervention (EMM=11.2%). Similar trends were seen for both pain instruments. Pain was reduced in 94.5% (224/237) of assessments where parents undertook an intervention. In 75.1% (178/237) of assessments indicative of pain, the score changed from pain to no pain, with PainChek Infant assessments more likely to report this change (odds ratio 4.1, 95% CI 1.4-12.3) compared with ObsVAS assessments.

**Conclusions:**

The use of standardized pain assessment instruments by parents at home to assess pain in their infants can inform their decision-making regarding pain identification and management, including determining the effectiveness of the chosen intervention. In addition to the construct validity and clinical utility of PainChek Infant and ObsVAS in this setting, feeding alone and a combination of feeding with medication use were the key pain intervention strategies used by parents.

## Introduction

Surgical procedures represent a well-established cause of pain in infants [1]. Good management of pain following various medical procedures, including same-day surgery, is important in infants, considering both short-term and long-term consequences that can arise as a result of a lack of neuronal pathway maturity [2-4]. Literature data suggest that early pain experiences cause activity-induced alterations in pain sensitivity, and these changes continue beyond infancy [3]. In this regard, it is worth noting that the long-lasting effects of suboptimal management of pain in children are more pronounced in comparison to the adult population [5]. Furthermore, in situations where children are exposed to repeated treatment and therefore repeated painful episodes, significant anxiety potentially leading to psychological and emotional consequences for the child and their carers can occur [6].

Despite compelling evidence suggesting the need to better recognize and manage pain in infants, pain in this population group still often remains underestimated and undertreated [4,7]. One of the key contributors to these issues is the fact that infants are unable to self-report pain due to their inability to verbally communicate, therefore making pain identification a major challenge in this population [4,8]. Eccleston et al [4] recently proposed 4 transformative aims with the view of improving pain management in children, one of which was that “pain should be made visible,” highlighting the need to adequately assess pain.

Circumcision is one of the most commonly performed same-day surgical procedures in the world [9,10]. It is estimated that the global prevalence of male circumcision is approximately 38%-39%, and depending on religious beliefs, these figures can reach over 95%, especially in countries with Muslim or Jewish majority [9]. Postoperative pain, such as that associated with circumcision surgery, is by definition acute pain, since it lasts for less than 3 months [4,11]. This pain involves nociceptive mechanisms, and its expected resolution usually occurs as a result of the healing process [4]. It has been suggested that following circumcision (same-day surgery), pain is persistent over a number of days after hospital discharge, which often makes treatment with analgesics necessary [12]. Other studies have also confirmed that children often experience moderate to severe levels of pain following same-day surgery [13-16]. Treatment of this pain generally takes place at home, considering that infants are usually discharged from the hospital on the same day the surgical procedure is performed and parents are then engaged in a number of demanding tasks, including pain assessment and treatment administration [14]. Various issues have been reported in relation to pain management by parents at home, and it has been suggested that the management of children’s postoperative pain at home, following hospital discharge, is generally poor [14,17]. The ability of parents to assess and identify pain and the need for assistance in this aspect have been reported as areas that need to be addressed [14,17,18].

When it comes to pain identification, there are a number of pain assessment scales that have been developed to assess pain in infants and young children, but there is no gold standard [19]. Most of these tools have limited evidence regarding their validity and clinical utility, and they are based on observer identification and evaluation of specific biomarkers indicative of pain [7,20,21]. None of the currently available pain assessment scales use automation to assess pain, and they are all limited by user subjectivity during pain assessment. Moreover, although these tools are used and validated in clinical practice settings, they are usually not used by parents at home. Additionally, there is limited literature reporting the pain assessment–related outcomes of tools specifically designed for use in the infant population (parents conduct pain assessments and use pain assessment tools at home).

This study aimed to explore parents’ assessments of their infants’ pain at home using standardized pain assessment instruments and investigate what pharmacological or nonpharmacological interventions they chose to manage pain following same-day surgery (ie, circumcision). The focus was on exploring the construct validity and clinical utility of 2 pain assessment scales, namely PainChek Infant and Observer-Administered Visual Analog Scale (ObsVAS). PainChek Infant has been designed specifically for use in the infant population and is an example of an automated digital pain assessment scale that uses artificial intelligence (AI) to identify facial indicators of pain [8,22]. ObsVAS is an instrument that has been commonly used to assess and quantify pain and distress [23]. In this regard, we aimed to explore the presence and improvement of pain following different interventions, evaluate how these pain assessment tools track pain levels, and assess their diagnostic accuracy across potential different cutoff points following the intervention and assessment of pain by parents at home.

## Methods

### Study Design

This prospective study collected data from the parents of infant boys up to 12 months of age undergoing circumcision at Kavaja Hospital in Prishtina in Kosovo from January to December 2023. Infants were excluded from the study if they had any psychiatric or developmental disorders or physical conditions that may interfere with the standard care program, or if their surgeon deemed they should not participate in the study. After undergoing same-day circumcision surgery and following hospital discharge, consenting parents were recruited to conduct pain assessments at home when pain was suspected and then at 30-minute and 60-minute intervals after intervention for a period of up to 3 days after surgery. Parents conducted pain assessments using 1 of the 2 validated pain assessment instruments provided: PainChek Infant or ObsVAS.

### Postsurgery Pain Assessment

#### PainChek Infant

PainChek Infant is a class 1 medical device in the form of a mobile app, which has regulatory clearance for the assessment of procedural pain in Australia and Europe. Assessment takes only 3 seconds to complete [8,22]. PainChek Infant uses AI for the automated recognition and analysis of an infant’s face, allowing the detection of 6 facial action units (AUs) indicative of the presence of pain: AU4 (brow lowering), AU9 (wrinkling of the nose), AU15 (lip corner depression), AU20 (horizontal mouth stretch), AU25 (parting lips), and AU43 (eye closure). These facial actions represent specific muscle movements (contractions or relaxations) as classified by the Baby Facial Action Coding System (BabyFACS) [24]. Each of the 6 AUs is scored using a binary scale (0=absent, 1=present), yielding a total potential score of 6 for comparative analysis with ObsVAS scores (standardized to a percentage). PainChek Infant’s algorithm to detect the abovementioned AU codes using AI was created on trained images of infants undergoing immunization procedures and corresponding to the age group of infants recruited for this study [8]. Initially, independent coders trained in the facial action coding system analyzed labeled infant images in relation to the presence of AU codes of interest. These labeled images were then used in training the model that is integrated into the mobile app. Using separate independent training and validation datasets, 5-fold cross-validation was employed to create the AI model. The tool has been specifically designed to assess pain in infants (aged 1-12 months), taking into account the facial actions commonly associated with pain in this population. It should be emphasized that prior to assessing facial indicators of pain, the users of PainChek Infant are instructed to rule out other common causes of nonpain-related distress such as the child being hungry, thirsty, frightened, too hot, too cold, tired, or sleepy; requiring a nappy change; wanting comfort; or requiring burping (passing of wind). This functionality comes up as an alert before proceeding to facial assessment. Previous research has found good correlation of scores between these tools (*r*=0.88, 95% CI 0.85-0.90; *P*<.001) [8]. However, the focus of this study was on evaluating the use of these tools by parents at home. Furthermore, high accuracy of PainChek Infant, with areas under the curve of 0.964 (standard mode) and 0.966 (adaptive mode), was shown [22]. PainChek Infant was also previously shown to perform well across various feasibility components [22].

#### ObsVAS Tool

ObsVAS is a tool that is commonly used to measure and quantify pain and distress [8,23]. The scale consists of a 100-mm line. In this line, 0 mm represents no pain or distress and 100 mm represents the worst possible pain or distress. It has been reported that VAS has good to excellent intrarater reliability and strong criterion validity [23]. Additionally, like PainChek Infant, ObsVAS was also previously shown to have good responsiveness in relation to the change of pain scores following a painful procedure [8].

#### Parent Education and Pain Assessment Diary

All parents who were using an iOS mobile device were approached to use the PainChek Infant app and were also trained on its use before their child was discharged from the hospital. Other parents who did not use iOS devices were approached to use the ObsVAS instrument. All parents participating in this study and consenting to use one of the pain assessment tools at home to assess and monitor their child’s pain were educated by their doctor on pain management and use of the pain assessment tool provided. As part of the training process, the correct use of both tools was demonstrated by a research assistant, who was a medical doctor by training and was specifically trained and competent in the use of both tools as well as the protocol of the study. When completing pain assessments at home, parents were also asked to record results in a diary. This was also covered in the training process. In relation to ObsVAS, parents were instructed to mark on the 100-mm line the score that corresponded to their perceived assessment of their child’s pain. Considering that PainChek Infant is a digital assessment tool, its results were also recorded automatically and synchronized via a cloud transmission system. For both tools, parents were instructed to perform a pain assessment when they suspected their child was in pain. When conducting a pain assessment, parents were instructed to record the results following a pain assessment in the diary in the morning (between 8:00 AM and 12:00 PM), afternoon (between 12:01 PM and 6:00 PM), or evening (after 6:00 PM), or when required if the assessment was outside of those timeframes. If an intervention was administered (pharmacological or nonpharmacological), parents were asked to record the intervention and then also perform further assessments 30 minutes and 60 minutes after the intervention. Parents were educated by the surgeons on pain intervention and management and were instructed to follow their doctors’ recommendations. Additionally, parents were informed that the results from the pain assessment instrument should not be used solely to decide whether to use analgesic therapy or to determine the required analgesic dosing and that this should be based on their doctors’ instructions. Moreover, they were told to consult with the doctor if they were not sure. Decisions undertaken by parents to manage postsurgical pain included medication administration (paracetamol or ibuprofen), feeding (breastfeeding, formula, or food), consoling (nursing or toys), and no action. These broad actions were also reclassified as no intervention, medicinal intervention, or nonmedicinal intervention.

### Data Analysis

Analysis was conducted using IBM SPSS (version 29) unless otherwise stated. Parents’ education levels were described using frequency (n) and percentage (%). Infant age was recorded as the age at admission for the circumcision procedure and was recorded as weeks or months. For 10 infants, the age recorded in weeks was converted to months by multiplying weeks by 0.23. Infant age was described using mean, SD, median, and 25th to 75th IQR. Normality was assessed using the Shapiro-Wilk test with age found not to be normally distributed, and appropriate nonparametric tests were applied. The Mann-Whitney *U* test examined age between pain assessment tool groups (ObsVAS versus PainChek Infant), with the standardized test statistic reported.

The chi-square analysis (or Fisher-Freeman-Halton exact chi-square test where cell counts <5 with exact 2-sided *P* values are reported) was undertaken to compare differences between the intervention used (none, medicinal, or nonmedicinal) and pain presence (absent or present). The test was conducted for the full sample and separately for PainChek Infant and ObsVAS. Binary logistic generalized estimating equations (GEEs) were used to examine if pain was present and assess the likelihood of an intervention occurring (Wald *χ^2^* and *P* value reported), with parameter-estimated odds ratios (ORs; Exp(β)), 95% Wald CIs for Exp(β), and *P* values reported for medicinal or nonmedicinal intervention compared to no intervention. The model accounted for individuals with repeated measurements and the within-subject variable of time. The confounding effects for age, instrument, and parent education were each examined separately in the basic model, with Quasi-likelihood under the Independence Model Criterion (QIC) goodness of fit indices used to compare models, and a lower number was associated with a better model fit [25]. The GEE method enables regression estimates when analyzing repeated measures with no assumption of the distribution of the response outcome [26,27].

Construct validity was assessed in several ways. First, GEE with standardized pain score as the outcome was evaluated with instrument, intervention, and age as fixed effects, accounting for individuals with repeated measurements and the within-subject variable of time. Model residuals (Q-Q plots) were visually assessed and assumptions were met. Estimated marginal means (EMMs) with 95% CIs were calculated, with Wald *χ^2^* and *P*-value model effects reported and Bonferroni-corrected pairwise comparisons for instrument and intervention undertaken. Separate models were also analyzed for each instrument on their original measurement scale. Second, we evaluated if pain scores improved after intervention (medicinal, nonmedicinal, or no intervention), with improvement categorized in 2 ways for analysis: pain relief and general pain reduction. For pain relief, a binary (Y/N) response to “did the pain score reduce to no pain” (PainChek Infant <2, ObsVAS=0) was used. For pain reduction, three groups were calculated: (1) no change or worse standardized pain score; (2) small standardized pain score improvement (≤33%); and (3) clinically important pain score improvement (>33%), standardized pain score returned to no pain (PainChek Infant ≤33%, ObsVAS=0%), or pain resolved (no further assessment undertaken at the final recorded timepoint, either 30 or 60 minutes). Receiver operating characteristic (ROC) analysis with sensitivity (true positive rate) and 100-specificity (false positive rate) was conducted using NCSS software (v21.0.14 2021) to assess whether there was a specific pain score where an intervention (generally) or medical intervention (specifically) was undertaken. Youden Index was used to determine the diagnostic accuracy across potential cutoff points (sensitivity + specificity – 1). The summary of area under the curve (AUC) scores overall and for each instrument has been reported. Each time period was a separate data entry for individuals.

Sensitivity analysis was conducted on baseline characteristics (age and education level) between parents who collected pain data versus those who did not. A Kruskal-Wallis test examined between-group differences for age (test statistic reported), while a Pearson chi-square (*χ^2^*) (Fisher-Freeman-Halton exact [cell counts <5]) test examined differences for education level. Two-sided *P* values have been reported throughout.

### Ethical Considerations

This study was approved by the research ethics committee of the Faculty of Medicine, University of Prishtina (approval number: 4860/22).

## Results

### Participant Characteristics

Parents of 109 infant boys undergoing circumcision were recruited in the study before the circumcision procedure and completed baseline measures (infant age and parent education). After surgery, 40 (36.7%) participants did not take any further part in the study (5 assigned to the ObsVAS group and 35 assigned to the PainChek Infant group). Sensitivity analysis did not detect any differences in age across participants who participated in postsurgery activities versus those who did not. A significant difference was detected in parent education, with those who did not return pain diaries coming from among university-educated parents (*χ^2^*_3_=12.0; *P*=.005). Of the remaining, 69 participants completed postsurgery pain assessments and returned their pain diaries. Of these, 24 (35%) were allocated to the ObsVAS group and 45 (65%) were allocated to the PainChek Infant group. Infants had a mean age of 5.1 months (SD 3.2 months; median 5.0 months, IQR 2.5-7.0 months). No significant difference in age was detected between the ObsVAS (mean 5.0, SD 3.6 months; median 4.0, IQR 2.0-7.5 months) and PainChek Infant (mean 5.1, SD 3.1 months; median 5.0, IQR 3.0-7.0 months) groups (*U*=–0.4; *P*=.68). Parents were predominantly university educated (50/69, 73%), with the remaining being high school educated or lower (19/69, 28%). There was no significant difference between the ObsVAS (high school: 7/24, 29%; university: 17/24, 71%) and PainChek Infant (high school: 12/45, 27%; university: 33/45, 73%) groups (*χ^2^*_1_=0.05; *P*>.99).

### Postsurgery Pain Presence and Pain Intervention Results

A summary of pain interventions administered after surgery over 3 days is provided in Table 1, with feeding alone and feeding with medication being the most common interventions reported by parents, followed by the use of medication alone. Parents also chose not to intervene. Among the medications administered, paracetamol was the most commonly reported, with ibuprofen used only in 3 instances.

Pain interventions were further described as no intervention, medicinal intervention, or nonmedicinal intervention (Tables 2-4). At baseline for each timepoint, a comparison was performed between pain present and absent and the pain intervention undertaken. Significant differences were only detected for day 2 afternoon and evening, and day 3 evening. For each, the absence of pain had a higher percentage of no intervention compared to the presence of pain, which had the lowest percentage of no intervention. Similar trends were seen when PainChek Infant and ObsVAS were examined separately. Further details are provided in Tables 2-4.

Pain presence and absence for the total sample and for the PainChek Infant and ObsVAS subsamples are reported in Table 5. From baseline assessments to 30-minute assessments and then 60-minute repeat assessments, there was a general reduction in the proportion of pain assessments, indicating the presence of pain, and there was a concomitant increase in those indicating no pain. A similar trend of decreasing pain presence versus pain absence was seen in assessments performed from day 1 to day 3. Some differences between PainChek Infant and ObsVAS were found across the time points, with PainChek Infant typically reporting a higher percentage of no pain and ObsVAS tending to report a higher percentage of pain present.

**Table 1:** Pain interventions administered across time after circumcision surgery.

Postsurgery time			Total, n		Intervention, n (%)								
					Consoling		Feeding only		Medications^a^		Medication and feeding		None
**Day 1**													
	Morning^b^	8		0 (0)		4 (50)		1 (13)		2 (25)		1 (13)	
	Afternoon^c^	52		1 (2)		18 (35)		19 (37)		12 (23)		1 (2)	
	Evening^d^	61		0 (0)		23 (38)		18 (30)		11 (18)		9 (15)	
	PRN^e^	14		0 (0)		5 (36)		1 (7)		3 (21)		5 (36)	
**Day 2**													
	Morning	62		0 (0)		28 (45)		11 (18)		10 (16)		13 (21)	
	Afternoon	61		2 (3)		34 (56)		6 (10)		6 (10)		13 (21)	
	Evening	56		0 (0)		26 (46)		6 (11)		6 (11)		18 (32)	
	PRN	8		0 (0)		1 (13)		0 (0)		3 (38)		4 (50)	
**Day 3**													
	Morning	40		0 (0)		19 (48)		6 (15)		3 (8)		12 (30)	
	Afternoon	40		0 (0)		17 (43)		1 (3)		5 (13)		17 (43)	
	Evening	33		1 (3)		13 (39)		2 (6)		5 (15)		12 (36)	
	PRN	9		0 (0)		5 (56)		0 (0)		1 (11)		3 (33)	

^a^Medications include paracetamol and ibuprofen pediatric formulations.

^b^Morning: 8:00 AM to 12:00 AM.

^c^Afternoon: 12:01 PM to 6:00 PM.

^d^Evening: after 6:00 PM.

^e^PRN: required basis outside of those timeframes.

**Table 2:** Interventions administered at baseline (1st measurement in the time period) for the overall sample.

Postsurgery time		Total, n	Total sample, n (%)				No pain^a^, n (%)				Pain^a^, n (%)				Pain group comparison^b^	
			None	Medicinal	Nonmedicinal	None		Medicinal	Nonmedicinal	None		Medicinal	Nonmedicinal	*χ*^*2*^ (*df*)		*P* value
**Day 1**																
	Morning^c^	8	1 (13)	3 (38)	4 (50)	1 (50)		0 (0)	1 (50)	0 (0)		3 (50)	3 (50)	3.2 (2)		.36
	Afternoon^d^	52	1 (2)	32 (62)	19 (37)	1 (11)		5 (56)	3 (33)	0 (0)		27 (63)	16 (37)	3.6 (2)		.23
	Evening^e^	61	9 (15)	29 (48)	23 (38)	5 (19)		9 (35)	12 (46)	4 (11)		20 (57)	11 (31)	3.1 (2)		.21
	PRN^f^	14	5 (36)	4 (29)	5 (36)	4 (67)		0 (0)	2 (33)	1 (13)		4 (50)	3 (38)	5.4 (2)		.10
**Day 2**																
	Morning	62	13 (21)	21 (34)	28 (45)	9 (32)		7 (25)	12 (43)	4 (12)		14 (41)	16 (47)	4.2 (2)		.11
	Afternoon	61	13 (21)	12 (20)	36 (59)	10 (36)		4 (14)	14 (50)	3 (9)		8 (24)	22 (67)	6.3 (2)		.047^g^
	Evening	56	18 (32)	12 (21)	26 (46)	17 (57)		1 (3)	12 (40)	1 (4)		11 (42)	14 (54)	24.4 (2)		<.001^g^
	PRN	8	4 (50)	3 (38)	1 (13)	4 (80)		1 (20)	0 (0)	0 (0)		2 (67)	1 (33)	4.7 (2)		.07
**Day 3**																
	Morning	40	12 (30)	9 (23)	19 (48)	10 (44)		3 (13)	10 (44)	2 (12)		6 (35)	9 (53)	5.5 (2)		.06
	Afternoon	40	17 (43)	6 (15)	17 (43)	14 (54)		2 (8)	10 (39)	3 (21)		4 (29)	7 (50)	5.0 (2)		.07
	Evening	33	12 (36)	7 (21)	14 (42)	9 (53)		1 (6)	7 (41)	3 (19)		6 (38)	7 (44)	6.3 (2)		.04^g^
	PRN	9	3 (33)	1 (11)	5 (56)	2 (50)		0 (0)	2 (50)	1 (20)		1 (20)	3 (60)	1.5 (2)		>.99

^a^PainChek Infant: ≤1=no pain, >1=pain; ObsVAS: 0=no pain, >0=pain.

^b^Fisher-Freeman-Halton exact chi-square test and exact 2-sided *P* value reported.

^c^Morning: 8:00 AM to 12:00 AM.

^d^Afternoon: 12:01 PM to 6:00 PM.

^e^Evening: after 6:00 PM.

^f^PRN: required basis outside of those timeframes.

^g^Statistically significant (*P*<.05).

**Table 3:** Interventions administered at baseline (1st measurement in the time period) for the PainChek Infant subsample.

Postsurgery time			Total, n		Total sample, n (%)							No pain^a^, n (%)							Pain^a^, n (%)							Pain group comparison^b^		
					None		Medicinal		Nonmedicinal		None			Medicinal		Nonmedicinal		None			Medicinal		Nonmedicinal		*χ*^*2*^ (*df*)			*P* value
**Day 1**																												
	Morning^c^	4		0 (0)		2 (50)		2 (50)		0 (0)			0 (0)		1 (100)		0 (0)			2 (67)		1 (33)		1.3 (2)			>.99	
	Afternoon^d^	31		1 (3)		16 (52)		14 (45)		1 (11)			5 (56)		3 (33)		0 (0)			11 (50)		11 (50)		2.6 (2)			.28	
	Evening^e^	40		4 (10)		22 (55)		14 (35)		3 (15)			8 (40)		9 (45)		1 (5)			14 (70)		5 (25)		3.6 (2)			.23	
	PRN^f^	5		2 (4)		0 (0)		3 (60)		2 (50)			0 (0)		2 (50)		0 (0)			0 (0)		1 (100)		0.8 (2)			>.99	
**Day 2**																												
	Morning	39		6 (15)		11 (28)		22 (56)		4 (19)			6 (29)		11 (52)		2 (11)			5 (28)		11 (61)		0.6 (2)			.90	
	Afternoon	39		8 (21)		9 (23)		22 (56)		6 (29)			4 (19)		11 (52)		2 (11)			5 (28)		11 (61)		1.8 (2)			.47	
	Evening	35		12 (34)		8 (23)		15 (43)		12 (50)			1 (4)		11 (46)		0 (0)			7 (64)		4 (36)		16.8 (2)			<.001^g^	
	PRN	3		2 (67)		1 (33)		0 (0)		2 (67)			1 (33)		0 (0)		0 (0)			0 (0)		0 (0)		—^h^			—	
**Day 3**																												
	Morning	25		7 (28)		4 (16)		14 (56)		6 (38)			2 (13)		8 (50)		1 (11)			2 (22)		6 (67)		2.1 (2)			.44	
	Afternoon	24		11 (46)		3 (13)		10 (42)		9 (50)			1 (6)		8 (44)		3 (33)			2 (33)		2 (33)		2.8 (2)			.27	
	Evening	20		6 (30)		4 (20)		10 (50)		4 (36)			1 (9)		6 (55)		2 (22)			3 (33)		4 (44)		1.8 (2)			.60	
	PRN	3		1 (33)		0 (0)		2 (67)		1 (33)			0 (0)		2 (67)		0 (0)			0 (0)		0 (0)		—			—	

^a^PainChek Infant: ≤1=no pain, >1=pain; ObsVAS: 0=no pain, >0=pain.

^b^Fisher-Freeman-Halton exact chi-square test and exact 2-sided *P* value reported.

^c^Morning: 8:00 AM to 12:00 AM.

^d^Afternoon: 12:01 PM to 6:00 PM.

^e^Evening: after 6:00 PM.

^f^PRN: required basis outside of those timeframes.

^g^Statistically significant (*P*<.05).

^h^Not applicable (no statistics computed as 1 pain group had no cases).

**Table 4:** Interventions administered at baseline (1st measurement in the time period) for the Observer-Administered Visual Analog Scale subsample.

Postsurgery time			Total, n		Total sample, n (%)							No pain^a^, n (%)							Pain^a^, n (%)							Pain group comparison^b^		
					None		Medicinal		Nonmedicinal		None			Medicinal		Nonmedicinal		None			Medicinal		Nonmedicinal		*χ*^*2*^ (*df*)			*P* value
**Day 1**																												
	Morning^c^	4		1 (25)		1 (25)		2 (50)		1 (100)			0 (0)		0 (0)		0 (0)			1 (33)		2 (67)		3.1 (2)			.50	
	Afternoon^d^	21		0 (0)		16 (76)		5 (24)		0 (0)			0 (0)		0 (0)		0 (0)			16 (76)		5 (24)		—^e^			—	
	Evening^f^	21		5 (24)		7 (33)		9 (43)		2 (33)			1 (17)		3 (50)		3 (20)			6 (40)		6 (40)		1.2 (2)			.58	
	PRN^g^	9		3 (33)		4 (44)		2 (22)		2 (100)			0 (0)		0 (0)		1 (14)			4 (57)		2 (29)		3.8 (2)			.11	
**Day 2**																												
	Morning	23		7 (30)		10 (44)		6 (26)		5 (71)			1 (14)		1 (14)		2 (13)			9 (56)		5 (31)		7.1 (2)			.03^h^	
	Afternoon	22		5 (23)		3 (14)		14 (64)		4 (57)			0 (0)		3 (43)		1 (7)			3 (20)		11 (73)		6.2 (2)			.04^h^	
	Evening	21		6 (29)		4 (19)		11 (52)		5 (83)			0 (0)		1 (17)		1 (7)			4 (27)		10 (67)		10.4 (2)			.004^h^	
	PRN	5		2 (40)		2 (40)		1 (20)		2 (100)			0 (0)		0 (0)		0 (0)			2 (67)		1 (33)		4.0 (2)			.20	
**Day 3**																												
	Morning	15		5 (33)		5 (33)		5 (33)		4 (57)			1 (14)		2 (29)		1 (13)			4 (50)		3 (38)		3.5 (2)			.30	
	Afternoon	16		6 (38)		3 (19)		7 (44)		5 (63)			1 (13)		5 (25)		1 (13)			2 (25)		5 (63)		4.1 (2)			.14	
	Evening	13		6 (46)		3 (23)		4 (31)		5 (83)			0 (0)		1 (17)		1 (14)			3 (43)		3 (43)		5.9 (2)			.06	
	PRN	6		2 (33)		1 (17)		3 (50)		1 (100)			0 (0)		0 (0)		1 (20)			1 (20)		3 (60)		2.5 (2)			.50	

^a^PainChek Infant: ≤1=no pain, >1=pain; ObsVAS: 0=no pain, >0=pain.

^b^Fisher-Freeman-Halton exact chi-square test and exact 2-sided *P* value reported.

^c^Morning: 8:00 AM to 12:00 AM.

^d^Afternoon: 12:01 PM to 6:00 PM.

^e^Not applicable (no statistics computed as 1 pain group had no cases).

^f^Evening: after 6:00 PM.

^g^PRN: required basis outside of those timeframes.

^h^Statistically significant (*P*<.05).

**Table 5:** Pain results across time points for the total sample and the tool subsamples.

Sample and postsurgery time				Baseline						30 minutes							60 minutes				
				n		No pain^a^, n (%)		Pain^a^, n (%)		n		No pain^a^, n (%)		Pain^a^, n (%)		n			No pain^a^, n (%)		Pain^a^, n (%)
**Total sample**																					
	**Day 1**																				
		Morning^b^	8		2 (25)		6 (75)		3		0 (0)		3 (100)		3			1 (33)		2 (67)	
		Afternoon^c^	52		9 (17)		43 (83)		36		16 (44)		20 (56)		27			16 (59)		11 (41)	
		Evening^d^	61		26 (43)		35 (57)		40		25 (63)		15 (38)		29			17 (59)		12 (41)	
		PRN^e^	14		6 (43)		8 (57)		10		6 (60)		4 (40)		8			6 (75)		2 (25)	
	**Day 2**																				
		Morning	62		28 (45)		34 (55)		31		22 (71)		9 (29)		27			21 (78)		6 (22)	
		Afternoon	62		29 (47)		33 (53)		29		23 (79)		6 (21)		24			20 (83)		4 (17)	
		Evening	56		30 (54)		26 (46)		33		23 (70)		10 (30)		27			24 (89)		3 (11)	
		PRN	8		5 (63)		3 (38)		5		3 (60)		2 (40)		4			3 (75)		1 (25)	
	**Day 3**																				
		Morning	41		24 (59)		17 (42)		23		18 (78)		5 (22)		17			14 (82)		3 (18)	
		Afternoon	40		26 (65)		14 (35)		19		13 (68)		6 (32)		16			12 (75)		4 (25)	
		Evening	33		17 (52)		16 (49)		18		12 (67)		6 (33)		15			11 (73)		4 (27)	
		PRN	9		4 (44)		5 (56)		6		3 (50)		3 (50)		5			3 (60)		2 (40)	
**PainChek Infant subsample**																					
	**Day 1**																				
		Morning	4		1 (25)		3 (75)		3		0 (0)		2 (100)		1			1 (100)		0 (0)	
		Afternoon^f^	31		9 (29)		22 (71)		19		10 (53)		9 (47)		12			9 (75)		3 (25)	
		Evening^g,h^	40		20 (50)		20 (50)		24		21 (88)		3 (13)		16			13 (81)		3 (19)	
		PRN	5		4 (80)		1 (20)		3		3 (100)		0 (0)		3			3 (100)		0 (0)	
	**Day 2**																				
		Morning^g^	39		21 (54)		18 (46)		18		16 (89)		2 (11)		16			14 (88)		2 (13)	
		Afternoon^g^	39		21 (54)		18 (46)		18		17 (94)		1 (6)		15			14 (93)		1 (7)	
		Evening^f^	35		24 (69)		11 (31)		21		15 (71)		6 (29)		16			15 (94)		1 (6)	
		PRN	3		3 (100)		0 (0)		2		2 (100)		0 (0)		2			2 (100)		0 (0)	
	**Day 3**																				
		Morning	25		16 (64)		9 (36)		14		13 (93)		1 (7)		11			10 (91)		1 (9)	
		Afternoon	24		18 (75)		6 (25)		11		8 (73)		3 (27)		10			8 (80)		2 (20)	
		Evening^g^	20		11 (55)		9 (45)		11		10 (91)		1 (9)		10			9 (90)		1 (10)	
		PRN^f^	3		3 (100)		0 (0)		2		2 (100)		0 (0)		2			2 (100)		0 (0)	
**ObsVAS^i^ subsample**																					
	**Day 1**																				
		Morning	4		1 (25)		3 (75)		1		0 (0)		1 (100)		2			0 (0)		2 (100)	
		Afternoon^f^	21		0 (0)		21 (100)		17		6 (35)		11 (65)		15			7 (47)		8 (53)	
		Evening^g,h^	21		6 (29)		15 (71)		16		4 (25)		12 (75)		13			4 (31)		9 (69)	
		PRN	9		2 (22)		7 (78)		7		3 (43)		4 (57)		5			3 (60)		2 (40)	
	**Day 2**																				
		Morning^g^	23		7 (30)		16 (70)		13		6 (25)		7 (54)		11			7 (64)		4 (36)	
		Afternoon^g^	23		8 (35)		15 (65)		11		6 (55)		5 (46)		9			6 (67)		3 (33)	
		Evening^f^	21		6 (29)		15 (71)		12		8 (67)		4 (33)		11			9 (82)		2 (18)	
		PRN	5		2 (40)		3 (60)		3		1 (33)		2 (67)		2			1 (50)		1 (50)	
	**Day 3**																				
		Morning	16		8 (50)		8 (50)		9		5 (56)		4 (44)		6			4 (67)		2 (33)	
		Afternoon	16		8 (50)		8 (50)		8		5 (63)		3 (38)		6			4 (67)		2 (33)	
		Evening^g^	13		6 (46)		7 (54)		7		2 (29)		5 (71)		5			2 (40)		3 (60)	
		PRN^f^	6		1 (17)		5 (83)		4		1 (25)		3 (75)		3			1 (33)		2 (67)	

^a^PainChek Infant: ≤1=no pain, >1=pain; ObsVAS: 0=no pain, >0=pain.

^b^Morning: 8:00 AM to 12:00 AM.

^c^Afternoon: 12:01 PM to 6:00 PM.

^d^Evening: after 6:00 PM.

^e^PRN: required basis outside of those timeframes.

^f^Statistically significant difference between PainChek Infant and ObsVAS for the absence and presence of pain at baseline (chi-square Fisher exact test 2-sided *P* value).

^g^Statistically significant difference between PainChek Infant and ObsVAS for the absence and presence of pain at 30 minutes (chi-square Fisher exact test 2-sided *P* value).

^h^Statistically significant difference between PainChek Infant and ObsVAS for the absence and presence of pain at 60 minutes (chi-square Fisher exact test 2-sided *P* value).

^i^ObsVAS: Observer-Administered Visual Analog Scale.

Regarding the association between pain and intervention, the basic GEE model found that when pain was present, an intervention was likely (*χ^2^*_2_=21.4; *P*<.001; QIC=550.5). Specifically, medicinal intervention had the highest odds (OR) of 12.6 (95% CI 4.3-37.0; *P*<.001), followed by nonmedicinal intervention with an OR of 5.2 (95% CI 1.8-14.6; *P*=.002) compared to no intervention when pain was present. The inclusion of a pain instrument in the basic model improved model fit (QIC=513.5), with both intervention (*χ^2^*_2_=27.5; *P*<.001) and instrument (*χ^2^*_1_=13.10; *P*<.001) being significantly associated with a pain outcome. Higher odds were reported for medicinal intervention (OR 17.5, 95% CI 5.9-51.8; *P*<.001) and nonmedicinal intervention (OR 7.3, 95% CI 2.4-22.1; *P*<.001) compared to no intervention when pain was present. ObsVAS had a higher odds of pain present (OR 4.4, 95% CI 2.0-9.9) compared to PainChek Infant. A model with an interaction term between intervention and instrument did not improve model fit (QIC=517.3), and the interaction term was not statistically significant (*χ^2^*_2_=2.1; *P*=.36).

The addition of age to the model improved model fit (QIC=510.9); however, age was not a significant effect (*χ^2^*_1_=2.7; *P*=.10), with both intervention (*χ^2^*_2_=30.3; *P*<.001) and instrument (*χ^2^*_1_=12.1; *P*<.001) being significantly associated with a pain outcome. Slightly higher odds were reported for medicinal intervention (OR 19.2, 95% CI 6.6-56.1; *P*<.001), and nonmedicinal intervention was more likely to occur (OR 6.9, 95% CI 2.3-20.6; *P*<.001) than no intervention. ObsVAS had higher odds of pain present (OR 4.3, 95% CI 1.9-9.9) compared to PainChek Infant. The addition of parent education to the model did not improve model fit (QIC=517.5), with it not having a significant effect (*χ^2^*_1_=0.1 *P*=.75).

**Figure 1:**
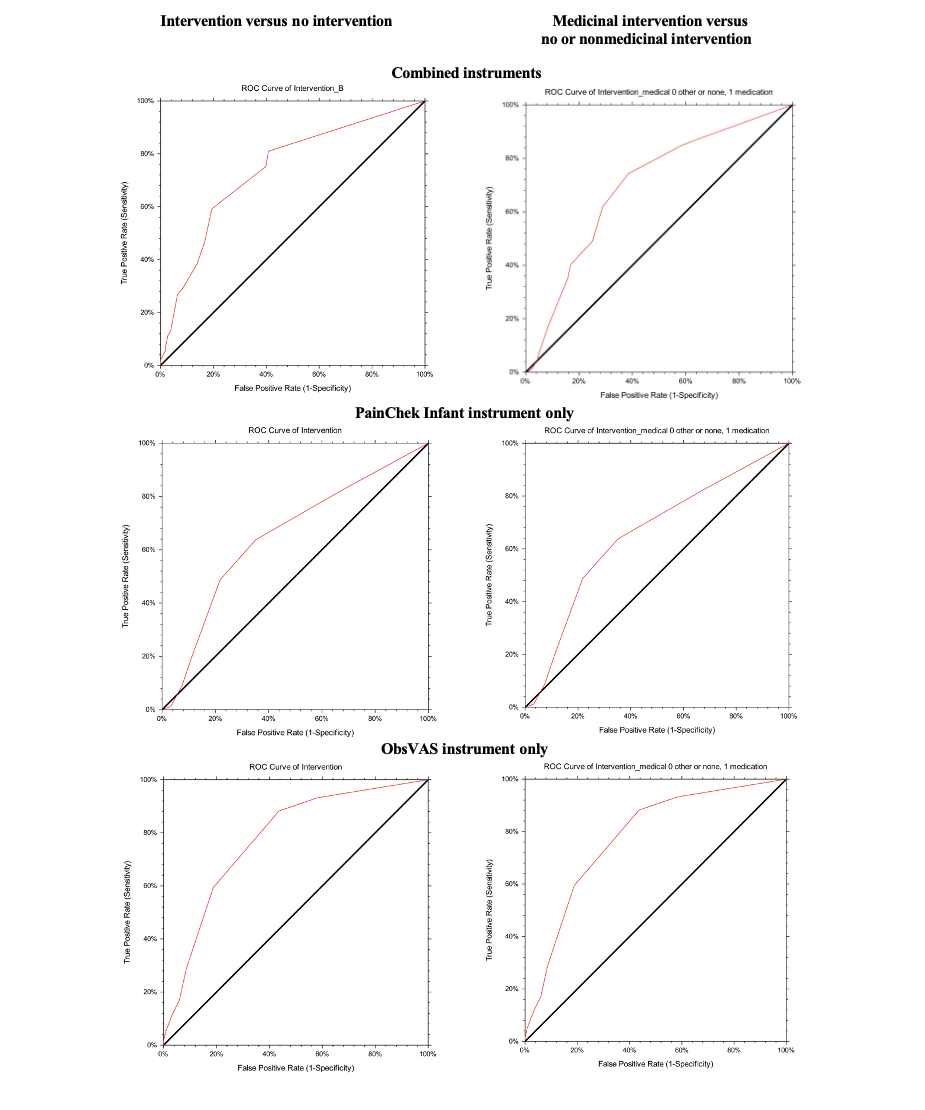
Sensitivity and specificity receiver operating characteristic (ROC) curve for the pain score with an intervention.

ROC analysis results are summarized in Table 6, and ROC curves are depicted in Figure 1. Youden Index determined cutoff values for the PainChek Infant and ObsVAS instruments and reported respective cutoff points for intervening values of ≥2 and ≥20 for intervention (medicinal and nonmedicinal) versus no intervention and similarly for medicinal intervention versus no intervention or nonmedicinal intervention. The combination of pain instruments using the standardized pain score Youden Index determined cutoff values of ≥10 for intervention (medicinal and nonmedicinal) versus no intervention; however, it reported values of ≥20 for medicinal intervention versus no intervention or other intervention, indicating a higher pain threshold for medicinal intervention to occur.

Evidence for construct validity assessed via GEE for standardized pain score reported a significant effect for instrument (*χ^2^*_1_=7.2; *P*=.007) and intervention (*χ^2^*_2_=43.4; *P*<.001) but not age (*χ^2^*_1_=1.9; *P*=.17). Pain scores were higher for PainChek Infant (EMM 27.5, 95% CI 22.6-32.3) compared to ObsVAS (EMM 18.5, 95% CI 14.2-22.9). Pain scores were the highest when a medicinal intervention was undertaken (EMM 34.2), followed by a nonmedicinal intervention (EMM 23.5), and were the lowest for no intervention (EMM 11.25). All Bonferroni-corrected intervention pairwise comparisons were significant (Table 7). Similar trends were seen for individual instrument models of PainChek Infant and ObsVAS, with EMM summarized by intervention presented in Table 7. It was noted that Bonferroni-corrected comparisons did not reach significance between medicinal and nonmedicinal interventions (*P*=.20) for the PainChek Infant instrument, and between no intervention and nonmedicinal intervention (*P*=.17) for the ObsVAS instrument.

For a subset of assessments indicative of pain (n=237), we assessed if the pain was relieved (ie, scores reduced to “no pain” levels). Of these, 178 (75.1%) infants recorded a change from pain to no pain. In this group recording an improvement to no pain, 37.6% (n=67) received medicinal intervention, 50.6% (n=90) received nonmedicinal intervention, and 8.9% (n=21) received no intervention. The instruments were also considered separately. For PainChek Infant (subset n=114) regarding improvement to no pain, 42.4% (n=42) received a medicinal intervention, 48.5% (n=48) received a nonmedicinal intervention, and 9.1% (n=9) received no intervention. For ObsVAS (subset n=123) regarding improvement to no pain, 31.6% (n=25) received a medicinal intervention, 53.2% (n=42) received a nonmedicinal intervention, and 15.2% (n=12) received no intervention. GEE reported a significant effect for instrument (*χ^2^*_1_=6.32; *P*=.01) and intervention (*χ^2^*_2_=7.3; *P*=.03). The PainChek Infant instrument was more likely to report a change from pain to no pain (OR 4.1, 95% CI 1.4-12.3) compared to the ObsVAS instrument.

Further descriptive analysis was used for this subset of assessments where pain was detected (n=237) to assess general pain reduction. Of these initial assessments of pain, 224 (94.5%) were followed by a reduction in pain at 30 minutes or 60 minutes after intervention, with 13 (5.5%) resulting in no change or worse pain. These were observed equally between medicinal intervention (n=100, 44.7%) and nonmedicinal intervention (n=102, 45.5%), with 9.8% (n=22) improving with no intervention. Observationally, similar trends were seen for PainChek Infant and ObsVAS. For PainChek Infant (n=114), when an intervention was undertaken, 110 (96.5%) assessments reported improvement in pain, with 4 (3.5%) reporting no change or worse pain. These were observed equally between medicinal intervention (n=49, 44.5%) and nonmedicinal intervention (n=51, 46.4%), with 9.1% (n=10) indicating improvement with no intervention. For ObsVAS (n=123), when an intervention was undertaken, 114 (92.7%) assessments reported improvement in pain, with 9 (7.3%) reporting no change or worse pain. These were observed equally between medicinal intervention (n=51, 44.7%) and nonmedicinal intervention (n=51, 44.7%), with 10.5% (n=12) indicating improvement with no intervention.

**Table 6:** Area under the receiver operating characteristic curve data for the pain scores of the tools.

Statistical item	Intervention vs no intervention				Medicinal intervention vs no or nonmedicinal intervention		
	Total	PainChek^a^	ObsVAS^b,c^	Total		PainChek^a^	ObsVAS^b,c^
Total, n	444	268	176	444		268	176
Pain, n	336	80	59	139		80	59
Proportion, %	75.7	29.9	33.5	31.3		29.9	33.5
Youden Index cutoff value	≥10	≥2	≥20	≥20		≥2	≥20
Area under the ROC^d^ curve	0.74	0.65	0.78	0.70		0.65	0.78
Standard error	0.03	0.04	0.04	<0.01		0.04	0.04
95% CI lower	0.68	0.58	0.70	0.64		0.58	0.70
95% CI upper	0.79	0.72	0.84	0.74		0.72	0.84
z statistic^e^	8.9	4.2	7.8	7.5		4.2	7.8
*P* value^e^	<.001	<.001	<.001	<.001		<.001	<.001

^a^PainChek score presented according to the raw scale 0-6.

^b^ObsVAS: Observer-Administered Visual Analog Scale.

^c^ObsVAS score presented according to the raw scale 0-100.

^d^ROC: receiver operating characteristic.

^e^Null hypothesis area under the curve=0.5.

**Table 7:** Estimated marginal mean pain score from generalized estimating equation models assessing the pain over time and type of intervention undertaken.

Model^a^ and intervention			EMM^b^		SE^c^		95% Wald CI		
							Lower		Upper
**Combined instrument model (scaled scores)**									
	Medicinal intervention^d,e^	34.22		2.95		28.44		40.00	
	Nonmedicinal intervention^d,f^	23.50		2.49		18.62		28.38	
	No intervention^e,f^	11.25		2.42		6.50		16.00	
**PainChek model (original 1-6 scale)**									
	Medicinal intervention^e,f^	2.34		0.31		1.73		2.95	
	Nonmedicinal intervention^e^	1.69		0.21		1.28		2.10	
	No intervention^e,f^	0.86		0.17		0.53		1.19	
**ObsVAS^g^ model (original 1-100 scale)**									
	Medicinal intervention^d,e^	30.49		2.54		25.50		35.47	
	Nonmedicinal intervention^d^	17.82		2.72		12.48		23.16	
	No intervention^e^	8.37		4.39		–0.23		16.97	

^a^Covariates appearing in the model are fixed for age.

^b^EMM: estimated marginal mean.

^c^SE: standard error.

^d^Statistically significant Bonferroni-corrected comparison between medicinal intervention and nonmedicinal intervention (*P*<.05).

^e^Statistically significant Bonferroni-corrected comparison between medicinal intervention and no intervention (*P*<.05).

^f^Statistically significant Bonferroni-corrected comparison between nonmedicinal intervention and no intervention (*P*<.05).

^g^ObsVAS: Observer-Administered Visual Analog Scale.

## Discussion

### Principal Findings

In this study, we reported parental assessment and management of pain at home following same-day circumcision surgery after hospital discharge for up to 3 days. Our findings showed that parents usually decided to undertake an intervention when their child was in pain. In this regard, they most commonly opted to choose either feeding alone or using medication together with feeding. Paracetamol was mostly used by parents (as opposed to only 3 cases of ibuprofen use). These results are in line with what was recommended and prescribed by the operating surgeons and what is consistent with recommendations from drug monographs of these medications [28,29]. It was interesting to note that in a number of cases, parents chose not to intervene even when pain was identified. There may be a number of reasons for this, but it is worth noting that fear of using analgesics as well as misconceptions regarding analgesic use by parents have been previously reported in the literature [14,17,18,30]. Nonetheless, our findings confirm that there was a relationship between the presence and absence of pain when an intervention was undertaken by parents (ie, pain assessments with pain as a result, for both PainChek Infant and ObsVAS, had the lowest proportion of no intervention). While similar trends were observed in this regard for both tools throughout various pain assessment timeframes, some differences were identified for day 2 afternoon and evening.

Our findings suggest that both tools were able to demonstrate the effectiveness of the intervention used by parents considering that the number of assessments indicating no pain generally increased over time following the intervention (both the 30- and 60-minute time points) and throughout days 1 to 3. While, as expected, pain presence reduced over this timeframe, our findings are consistent with the results by Freeman et al [12], which suggested that pain is persistent for a few days following circumcision. In cases where there were significant differences between tools, our findings indicated that ObsVAS was associated with reporting a higher proportion of the presence of pain in comparison with PainChek Infant. When it comes to these instrument-specific differences, it should be noted that assessments with different instruments were not conducted on the same subjects. Additionally, PainChek Infant has a clear cutoff (≥2 out of 6) for pain as opposed to ObsVAS, which considers any score above 0 to indicate pain, and a low score in ObsVAS is not necessarily a clinically important score indicative of pain. While it was noted that the aggregated proportion of ObsVAS assessments indicative of pain was higher compared to PainChek Infant, it is important to note that when exploring threshold pain scores in relation to intervention, these were similar (ie, ≥20 out of 100 [20%] for ObsVAS and ≥2 out of 6 [33%] for PainChek Infant). Nonetheless, the determination of the cutoff score for PainChek Infant confirms previous results reported by Hughes et al [22], providing additional evidence on its pain score threshold and clinical utility. Previous validation of the tool was conducted using prerecorded videos of children undergoing immunization, and assessments were undertaken by trained assessors on those video recordings, as opposed to parents conducting assessments in a “real-life” environment at home [8,22]. Another key point of the difference between the instruments used is that PainChek Infant is AI-enabled and fully automated, while ObsVAS relies on the ability of parents to observe and then rate their child’s pain. In this regard, variability in parents’ judgment related to pain assessment and subjectivity when using observational scales have been reported previously [19,31,32]. Therefore, this should be taken into consideration when interpreting the results obtained when parents use ObsVAS.

Our findings, based on the GEE model, confirm the likelihood of parents undertaking an intervention when pain is identified with either PainChek Infant or ObsVAS. A medicinal intervention, which included pharmacological treatment (mainly paracetamol), was more likely during pain presence as opposed to a nonmedicinal intervention such as breastfeeding or comforting. Additionally, when pain was present, parents were more likely to select a nonmedicinal intervention as opposed to no intervention at all. Furthermore, for both instruments used, there was a clear relationship between higher pain scores and the choice of intervention, with the highest pain scores being more closely associated with medicinal intervention, followed by nonmedicinal intervention. No intervention category was associated with the lowest pain scores. These findings confirm the ability of parents to respond to the presence of pain in their child and to choose a treatment option to manage the pain. This was observed independent of parents’ education levels. Our findings confirm the effectiveness of the interventions undertaken by parents considering that the presence of pain, which was indicated by most assessments, improved to no pain. While this trend was observed for both instruments, the use of the PainChek Infant instrument was associated with a higher likelihood of change from pain to no pain compared to ObsVAS. Nonetheless, our results suggest that both instruments assisted parents in identifying pain and making decisions on pain management and helped in rechecking that the pain experienced by their child had diminished.

One of the key strengths of this study is that it provides insights into parents’ use of standardized pain assessment tools to identify and make decisions on the management of their child’s pain at home. There is scarce literature in this area. However, there are a number of limitations that should be considered overall and that should be taken into account when interpreting our findings. First, the study was conducted with a small number of parents at 1 hospital center only. The issues of small sample size and single-center design limit the generalizability of our findings to a broader audience of parents, and this should be considered when interpreting our findings. Furthermore, other hospitals may have different protocols for managing infant pain following minor surgical procedures, which may affect how parents behave during pain management at home. Second, while ObsVAS was only offered to parents who were using an Android device, it is difficult to judge how this group of parents would have behaved if they were iOS users. Previous studies have already reported on behavioral differences between Android and iOS users [33,34]. At the time when the study was conducted, PainChek Infant was only available on iOS. Moreover, it remains unclear why a higher proportion of those parents using an iPhone device (approached to use PainChek Infant) failed to complete the study despite follow-up attempts. Additionally, it should be noted that the majority of parents who participated in this study were female. The differences may have been driven by greater recruitment of parents using iOS devices, and female individuals have been reported to be more inclined to use iOS devices as opposed to Android devices [34]. Considering this, the recruitment of Android users may have resulted in a higher percentage of male participants conducting pain assessments, which may have impacted the study findings regarding intervention choices. This limitation suggests that there is a need for more research comparing differences between iOS and Android users, especially in the area of postsurgical infant care and specifically pain management. This was not a focus of our study. Another consideration regarding the interpretation of our study findings is parents’ education levels, with the majority of parents administering PainChek Infant and ObsVAS having completed tertiary-level education. Nonetheless, our results appear to not have been affected by this particular factor considering that our findings suggest that the ability of parents to respond to the presence of pain in their child was independent of their education. The potential overlap of pain- and nonpain-related distress among parents during the assessment of pain should also be considered. This is a potential limitation that has been acknowledged by a number of other studies reporting on the use of other pain assessment tools [8,35,36]. However, given that in both groups of parents (ie, PainChek Infant and ObsVAS users) the source of pain was known (ie, circumcision procedure), the possibility for this overlap was reduced. To further mitigate this issue, parents using PainChek Infant had the ability to rule out other common causes of nonpain-related distress, such as the child being hungry or tired, as this functionality is built into the PainChek Infant app itself. This could also explain some differences identified between the 2 instruments and may have affected parents’ decisions regarding the choice of intervention after ruling out nonpain-related distress. Further studies are needed to explore this issue. The study could have also benefited from the exploration of other available pain assessment tools used in infants, such as the Neonatal Facial Coding System. However, this decision was balanced against potential implications related to recruitment and the need for further training of parents. Further research in this area is recommended.

Although this study had the abovementioned limitations, it provided valuable insights into parents’ use of standardized pain assessment tools at home, the selection of interventions when pain is detected in their infants, and the effectiveness of these interventions. Additionally, the study provided further evidence regarding the construct validity and clinical utility of both PainChek Infant and ObsVAS, which were used by parents to assess pain at home for over 3 days after surgery. Both tools were able to inform clinical decision-making and were instrumental in determining the effectiveness of interventions chosen by parents.

### Conclusions

Our findings provide insights into parental assessment and management of pain in their infants at home following hospital discharge from same-day surgery (ie, circumcision). Feeding alone and a combination of feeding with medication use were the key pain intervention strategies used by parents after identifying pain using standardized pain assessment tools. We further demonstrated the construct validity and showed that PainChek Infant and ObsVAS have similar clinical utility in assisting parents regarding the selection of pain interventions and the determination of their effectiveness, thus supporting their clinical decision-making.

## Abbreviations

AI: artificial intelligence
AU: action unit
EMM: estimated marginal mean
GEE: generalized estimating equation
ObsVAS: Observer-Administered Visual Analog Scale
OR: odds ratio
QIC: Quasi-likelihood under the Independence Model Criterion
ROC: receiver operating characteristic
